# Supplementary material for: Virtual Screening and Binding Analysis of Potential CD58 Inhibitors in Colorectal Cancer (CRC)
Source: Molecules. 2023 Sep 27;28(19):6819. doi: 10.3390/molecules28196819 (PMC10574072; doi:10.3390/molecules28196819)
Supplement: Supplementary file 1 [file molecules-28-06819-s001.zip › molecules-2622537-supplementary.pdf]

Supplemental materials

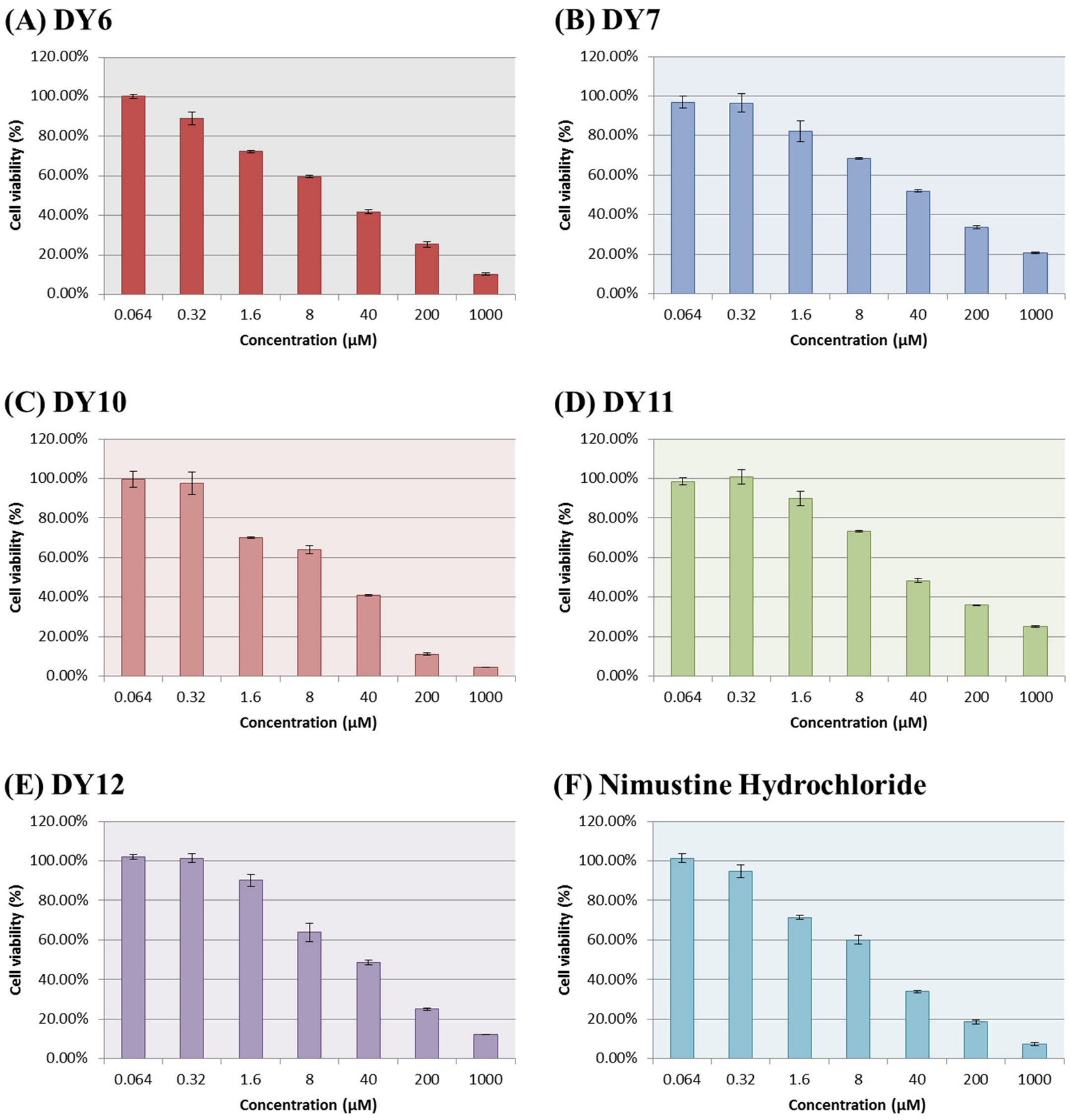

Figure S1. Potential anti-proliferation of DY6 (A), DY7 (B), DY10 (C), DY11 (D), DY12 (E) and Nimustine Hydrochloride (F) on SW620 cells. The cells were treated with different concentrations of compounds for 48h, and the cell viability was determined by CCK-8 assay. Each test was repeated three times, and the standard deviation was calculated.
